# Whole Genome Sequencing in Advanced Lung Cancer can be Performed Using Diff-Quik Cytology Smears Derived from Endobronchial Ultrasound, Transbronchial Needle Aspiration (EBUS TBNA)

**DOI:** 10.1007/s00408-023-00631-9

**Published:** 2023-07-05

**Authors:** David Fielding, Andrew J. Dalley, Mahendra Singh, Lakshmy Nandakumar, Vanessa Lakis, Haarika Chittoory, David Fairbairn, Kaltin Ferguson, Farzad Bashirzadeh, Michael Bint, Carl Pahoff, Jung Hwa Son, Alan Hodgson, John V. Pearson, Nicola Waddell, Sunil R. Lakhani, Gunter Hartel, Katia Nones, Peter T. Simpson

**Affiliations:** 1 Department of Thoracic Medicine, The Royal Brisbane & Women’s Hospital, Brisbane, Australia; 2 Faculty of Medicine, UQ Centre for Clinical Research, The University of Queensland, Brisbane, Australia; 3 Pathology Queensland, The Royal Brisbane & Women’s Hospital, Brisbane, Australia; 4 QIMR Berghofer Medical Research Institute, Brisbane, Australia; 5 Department of Thoracic Medicine, Sunshine Coast University Hospital, Birtinya, Australia; 6 Department of Respiratory Medicine, Gold Coast University Hospital, Southport, Australia; 7 School of Biomedical Sciences, The University of Queensland, Brisbane, Australia

**Keywords:** Cytology, Endobronchial ultrasound–guided transbronchial needle aspiration (EBUS TBNA), Lung cancer, Molecular diagnostics, Whole genome sequencing

## Abstract

**Introduction:**

Maximising alternative sample types for genomics in advanced lung cancer is important because bronchoscopic samples may sometimes be insufficient for this purpose. Further, the clinical applications of comprehensive molecular analysis such as whole genome sequencing (WGS) are rapidly developing. Diff-Quik cytology smears from EBUS TBNA is an alternative source of DNA, but its feasibility for WGS has not been previously demonstrated.

**Methods:**

Diff-Quik smears were collected along with research cell pellets.

**Results:**

Tumour content of smears were compared to research cell pellets from 42 patients, which showed good correlation (Spearman correlation 0.85, *P* < 0.0001). A subset of eight smears underwent WGS, which presented similar mutation profiles to WGS of the matched cell pellet. DNA yield was predicted using a regression equation of the smears cytology features, which correctly predicted DNA yield > 1500 ng in 7 out of 8 smears.

**Conclusions:**

WGS of commonly collected Diff-Quik slides is feasible and their DNA yield can be predicted.

## Introduction

When a tissue diagnosis of lung cancer is made the samples must be simultaneously used for immunohistochemical subtyping and molecular genetic testing to assess for the presence of actionable mutations. Endobronchial ultrasound-guided, transbronchial needle aspiration (EBUS TBNA) is a common procedure to make the tissue diagnosis of advanced lung cancer [1, 2]. Small amounts of material from fine needle aspirates are deposited on smears for microscopy in the procedure room (Diff-Quik smears) and for formal microscopy (pap smears), while the majority of the sample is collected to make formalin-fixed paraffin-embedded (FFPE) cell blocks [3, 4]. FFPE cell blocks from EBUS TBNA may have adequate tumour content for molecular analysis in as few as 43% of samples [5]. Regarding next generation panel sequencing success rates for EBUS TBNA acquired FFPE cell blocks range from 60 to 93% [6–10]. Conversely Diff-Quik smears contain cancer cells in over 90% of lung cancer patients [11] and are typically never required after their use in the procedure room [12]. These smears have great potential for molecular testing. They allow a rapid estimation of tumour cell content and avoid the impact of formalin on DNA [12]. We and others have shown > 95% success of sequencing of these smears, including with large sequencing panels [12–14]. Using smears for sequencing preserves the FFPE cellblock for immunohistochemistry and other developing spatial techniques [15].

In this brief report we take Diff-Quik smears further by exploring their potential for whole genome sequencing (WGS). WGS is not standard of care at this time, however with progressively falling costs it could be more widely used in the clinic [16–18]. WGS can detect all forms of molecular abnormalities including point mutations, fusion genes, and chromosomal damage [19], indicating it can detect all actionable mutation types in lung cancer. Further, WGS future-proofs the need to incrementally expand the size of molecular panels.

Successful performance of WGS testing of Diff-Quik smears is highly dependent on the tumour content of the sample, with the proportion of malignant cells preferably to be > 40% and the slide to yield > 1500 ng DNA. We recently showed that over a third of smears had > 1500 ng DNA and all of which had > 40% tumour cellularity [12], suggesting WGS is potential feasibility for a significant number of Diff-Quik samples. Further, it is important to show that the Diff-Quick smear is representative of the FFPE cell block, such that they demonstrate similar tumour content, especially when considering the cell blocks are representative of multiple needle aspirates from the same lymph node, whereas each smear represents just one needle pass.

In demonstrating feasibility of Diff-Quik smears for WGS, we therefore sought to determine (i) whether Diff-Quik smears could yield sufficient tumour content compared with matched research cell pellets; (ii) whether we can predict the amount of DNA on a smear using a simple set of microscopy criteria [12] prior to attempting WGS; and (iii) whether Diff-Quik smears could represent the sole source of WGS material when cell pellets do not yield sufficient material for sequencing.

## Methods

The samples were part of a large prospective study exploring the benefits of Diff-Quik smears (Institutional Review Board approval (HREC/17/QRBW/301); QIMR P2404) [20]. Patients with suspected lung cancer undergoing EBUS TBNA sampling had standard of care testing including Diff-Quik smears, PAP smears and FFPE cell blocks. Research samples were collected frozen or in RNALater for creation of cell pellets. Diff-Quik smears were evaluated by two cyto-pathologists to estimate the percent of malignant cells and overall malignant cell count on the slide. The smear evaluation process by the two cyto-pathologists took approximately 10 min per smear. In addition, smear area was measured from digital slide scans [15]. These 3 parameters were included in a lognormal regression [12] model to estimate DNA yield, as follows:

EXP([1.6 if malignant cells are ≥ 50% malignant cells] + [ 1.2 for malignant cell count estimate ≥ score 2 or 1000 cells] + [0.255* % slide area covered by smear] + 4.14) = ng DNA for that smear.

DNA was extracted from research cell pellets using the AllPrep DNA/RNA Mini Kit, from matched blood samples using the QiAmp DNA Blood Mini Kit, and from smears using the QiAmp DNA Micro Kit (Qiagen). DNA quantity and integrity were measured by Qubit Assay (Thermo Fischer) and TapeStation (Agilent). DNA from research pellets and Diff-Quik slides, with matched normal DNA, were analysed by Infinium Global Screening SNP arrays (Illumina) to determine the tumour content (% tumour) of the samples [21]. Eight smears with > 1ug of DNA and > 40% tumour content [22–24] estimated by SNP arrays underwent WGS: four smears had concomitant WGS from fresh cell pellet to allow comparison of genome data and four smears were selected to see if we could “rescue” cases where the fresh cell pellet was inadequate for WGS.

DNA from Diff-Quik smear, cell pellet and matched blood were subjected to WGS. Samples were sequenced to an average read depth of 36.9x (range 31.5 to 44.1) for blood and 70.3x (range of 50 to 77.5x) for tumour samples (smear or cell pellet). The detection of somatic mutations was performed as previously described [25, 26]. Results of standard of care pathology and molecular testing were recorded.

## Results

**Fig. 1:**
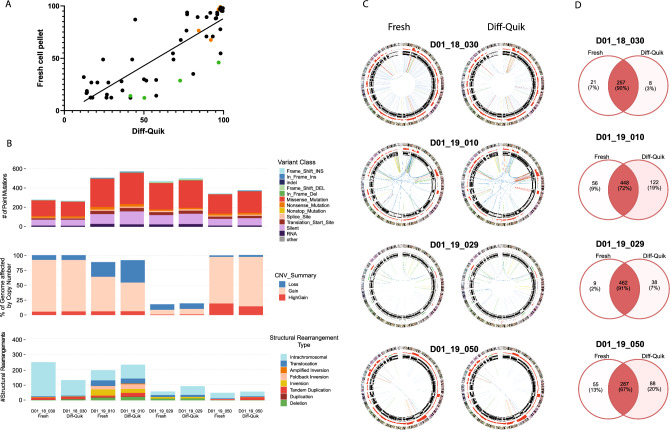
Comparison of Diff-Quik smears and fresh cell pellet samples obtained from the same EBUS TBNA procedures. **A** Tumour content estimated using SNP arrays for Diff-Quik samples and fresh cell pellets collected during the same EBUS TBNA procedure of advanced lung cancers. Spearman correlation = 0.85 (*p* < 0.0001). Orange dots are samples that both Diff-Quik smears and fresh cell pellet were subjected to whole genome sequencing. Green dots represent samples where only Diff-Quik smears were sequenced by WGS, as fresh samples had insufficient tumour content (< 40%) or DNA yield. **B** Number and distribution of tumour specific mutations, including point mutations, copy number changes and structural rearrangements detected in Diff-Quik smears and fresh cell pellets. **C** Circos plots for fresh cell pellets and Diff-Quik samples. Each plot shows chromosomes in the outer ring, followed by copy number alterations (green = loss, red = gain), inner ring represents B-allele frequency data which can be used to identify regions of loss of heterozygosity, lines in the middle of the circos plot indicate structural rearrangements. **D** Venn-diagrams show the overlap in somatic point mutations detected in Diff-Quik samples and fresh cell pellet from each patient

The tumour content was estimated by SNP arrays for 55 Diff-Quik smears and 44 fresh samples from 42 patients. Figure 1 shows comparisons of tumour content and sequencing of fresh tissue and Diff-Quik smears. Median DNA yield of these smears was 1,965 ng (range: 216–12,690 ng) and the median DNA integrity (DIN) was 4.3 (range: 1.9–6.6). Fresh sample results were 17,600 ng (range 434–19,6200 ng) and 6.8 DIN (range 1.8–8.8), respectively. Tumour content estimated by SNP arrays for the two sample types (research cell pellet and Diff-Quik) showed good correlation (Spearman correlation 0.85, p < 0.0001) (Fig. 1A).

Table 1 shows the clinical and sample information of the eight cases that underwent WGS, including the extracted DNA yield and quality, as well as the DNA yield predicted by cytopathology scores. In these 8 patients which underwent WGS the median number of TBNA needle passes overall was 4.0 ± 1.0. All the smears with actual DNA content > 1500 ng had predicted DNA values also of > 1500 ng. This value was selected as an approximate value of DNA required to perform DNA quality control by SNP array and then WGS. For case D01_18_034, the smear had a DNA yield of 1452 ng and a predicted yield of 252 ng; the discrepancy likely due to microscopy raw scores being at the low margin of the algorithm, in particular the smear surface area was only 7% of the slide area.

**Table 1:** Patient and sample details for cases subjected to WGS, including mutations reported by standard of care and by WGS

Cases	D01_18_030	D01_19_010	D01_19_029	D01_19_050	D01_18_034	D01_19_020	D01_19_046	D01_21_090
Clinical								
Age	56	53	61	60	75	74	66	67
Gender	M	F	F	F	F	F	M	M
Clinical staging	T2N3MX	T4M3M1a	TXN3M0	T2bN2M0	T4N3M0	T2bN3M0	T2aN3M0	T3NbM0
Tissue diagnosis	NSCLC	SCLC	SCLC	NSCLC	LUAC	LCNE	NSCLC	LUAC
Molecular SOC	ND (insufficient tissue)*	NI	NI	KRAS:p.Gly12Ala	Neg#	Neg	Neg#	ROS**
Type of testing	ND	NI	NI	Panel	Cast PCR	Panel	Cast PCR	Panel
PDL1	ND	NI	NI	20%	< 1%	ND	< 1%	70%

Research	DQ SMEAR	PELLET	DQ SMEAR	PELLET	DQ SMEAR	PELLET	DQ SMEAR	PELLET	DQ SMEAR	PELLET	DQSMEAR	PELLET	DQ SMEAR	PELLET	DQ SMEAR	PELLET
DNA Yield (ng)	3000	3200	4458	18,900	7920	191,800	9120	8740	1452	3540	30,048	730	1932	6800	3900	10,200
Predicted DNA yield (ng)	2795		5488		1947		2289		252		1702		2820		10,306	
SNP array tumour content	92	67	97	97	98	99	84	76	51	28(NS)	97	45(NS)	41	12(NS)	50	12(NS)
DIN	5.2	2.5	5.4	7.4	5.9	8.2	5.7	7.5	3.2	3.5	5.9	N/A	6.1	8.2	6.3	8.4
WGS mutations	KRAS p.Gly12Asp	KRAS p.Gly12Asp					KRAS p.Gly12Ala	KRAS p.Gly12Ala	STK11 p.Glu374*	NS		NS	STK11 p.Lys48fs*	NS	ROS1:CD74 Fusion	NS
	STK11 p.Glu120*	STK11 p.Glu120*														

*M* male, *F* female, *ND* Not Done, *NI* Not indicated, *SOC* Standard of care, *NS* No sequencing i.e. whole genome sequencing not performed due to low tumour content or DNA yield, *DIN* DNA integrity number, *SNP array* single nucleotide polymorphism arrays, *Panel* 22 gene targeted sequencing panel, *Cast PCR* EGFR hotspot testing, *NSCLC* Non-small cell lung carcinoma, not otherwise specified, *SCLC* small cell lung carcinoma, *LUAC* Lung adenocarcinoma, *LCNE* Large cell neuroendocrine carcinoma

*Insufficient tissue for molecular SOC testing; smear was suitable for sequencing, and tier 4 mutations detected (https://www.oncokb.org/)

^#^No mutation reported on SOC testing; smear was suitable for sequencing, and tier 4 mutations detected

**Confirmed by ROS1 immunohistochemistry on CT core biopsy prior to EBUS TBNA staging (which provided smear), and fluorescence in situ hybridisation revealed a rearrangement of the ROS1 locus (± loss of 5' signal) in 76% of tumour cells

Both smears and the research cell pellets obtained from the same procedure demonstrated good agreement in the number and type of somatic single nucleotide variants, copy number alterations, and structural rearrangements detected by WGS (Fig. 1B). Circos plots (Fig. 1C) illustrate the concordance in the pattern of structural alterations and Venn diagrams (Fig. 1D) indicates that the majority (67–91%) of point mutations detected in the freshly collected specimens were also detected in the diagnostic Diff-Quik smears.

**Fig. 2:**
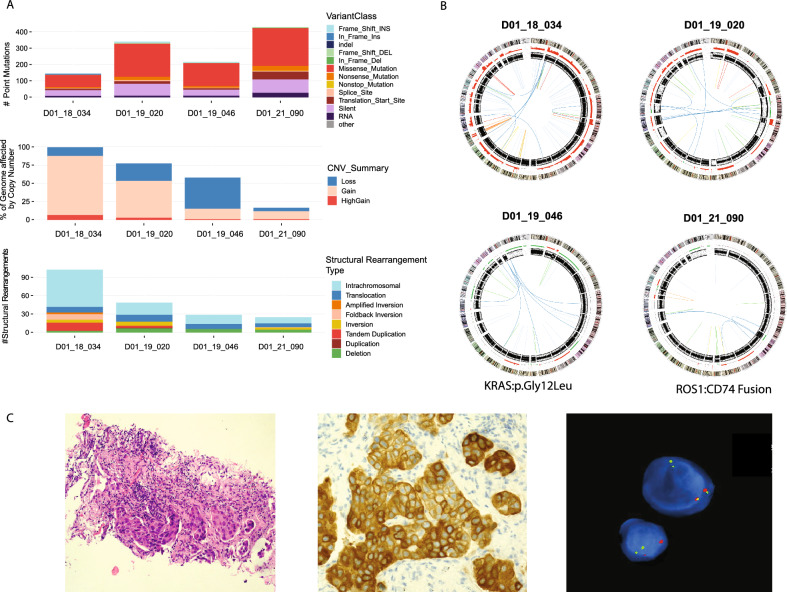
Somatic alterations detected in Diff-Quik smears from cases where the matched fresh cell pellet was not suitable for whole genome sequencing. **A** Number of point mutations and indels, percentage of the genomes affected by copy number, and the number and types of structural rearrangements. **B** Circos plots for individual cases, showing chromosomes in the outer ring, followed by copy number alterations (green = loss, red = gain), inner ring represents B-allele frequency data which can be used to identify regions of loss of heterozygosity, lines in the middle of the circos plot indicate structural rearrangements. **C** Images from diagnostic testing for case D01_21_090 (adenocarcinoma, CT core biopsy prior to EBUS TBNA staging), showing haematoxylin and eosin, ROS1 immunohistochemistry (H-score of 220) and *ROS1* FISH (Zytolight SPEC ROS1 (6q22.1) Dual Colour, Break Apart Rearrangement Probe). *ROS1* fusion positive cells were indicated by single red and green signals, as observed here

In four samples where the fresh cell pellet did not provide appropriate material for WGS, the smear samples were successfully sequenced (Fig. 2). The somatic mutations identified included reportable mutations revealed by standard of care testing, i.e. *KRAS*: c. 35G > C p. (Gly12Ala) in case D01_19_046 and a fusion between *ROS1* and *CD74* in case D01_21_090, which was reported diagnostically as a *ROS1* rearrangement by immunohistochemistry, and fluorescence in-situ hybridisation (Fig. 2C).

In three cases, extra mutations in *KRAS* and *STK11* (Tier 4 mutations) were detected by WGS (Table 1). Considering the added overall benefit, from the 8 smears there was added molecular information from this WGS single test in 4 patients (50%): 3 with tier 4 mutations and 1 with a ROS 1 fusion. These 4 include 1 patient where the standard of care cell block was “insufficient for testing.”

## Discussion

Others have demonstrated that WGS is feasible from EBUS-TBNA specimens [27]. Here we advance this knowledge to demonstrate for the first time the feasibility of conducting WGS on Diff-Quick smears. A common clinical problem arises when the FFPE cell block yields insufficient material for genomic testing; here we show that not only can Diff-Quik smears negate this problem, but also that they can yield comprehensive genomic data capturing multiple types of somatic mutations in one molecular test.

To demonstrate the utility of WGS, we observed good agreement between smear and fresh tissue WGS for point mutations (67 to 91% agreement), copy number alterations and structural rearrangements. Importantly, WGS identified Tier 1 somatic alterations reported by accredited methodology and Tier 4 mutations with potential relevance to future genomic based therapies. Further, smears permitted WGS for cases where the fresh tissue and/or FFPE cell pellets had insufficient material. In three NSCLC cases, smears provided sufficient material for comprehensive genomic testing where SOC testing was insufficient or had no reportable mutations.

Good correlation of tumour content between fresh cell pellets and smears gives confidence that a carefully selected Diff-Quik smear can represent an average of all needle passes made into the node.

Not all smears will have sufficient DNA (> 1500 ng) for WGS or have sufficient malignant cell content (> 40%). We suggest the use of our cytology-based prediction algorithm can assist in selecting smears that will yield sufficient DNA. Further this prediction could allow selection of the best of all the smears from a procedure for sequencing. The algorithm will continue to be improved with further samples from the clinic. Generally, our predictions under-estimated the actual yield of DNA obtained from the smears, however all smears with > 1500 ng DNA were correctly predicted above that threshold. Two cases significantly underestimated DNA content, as scores for estimating cell counts are arbitrarily set at a maximum of 4000 cells some smears clearly have many more cells than this. Some samples at the lower marginal end of the prediction algorithm may be excluded, but conversely the correct identification of samples with > 1500 ng can rule them in.

In our study, Diff-Quik smears were one or two years old, which might have impacted the DNA quality obtained. We would expect freshy collected smears to have DIN approaching that of fresh cell pellets.

Two smears were from patients with SCLC and were chosen for their tumour content to contribute to this proof-of-concept study. Presently there are no specific molecular targets for SCLC however WGS could have a future role in aiding treatment decision making in the future [28].
